# Factors affecting respiratory vaccination in Oklahoma cow-calf operations

**DOI:** 10.3389/fvets.2025.1502455

**Published:** 2025-04-25

**Authors:** Kristina Harwell, Amy D. Hagerman, Kellie Curry Raper, Hannah Shear, Rosslyn Biggs, Barry Whitworth

**Affiliations:** ^1^Simmons Pet Food, Siloam Springs, AR, United States; ^2^Department of Agricultural Economics, Oklahoma State University, Stillwater, OK, United States; ^3^College of Veterinary Medicine, Oklahoma State University, Stillwater, OK, United States; ^4^Oklahoma Cooperative Extension Service, Oklahoma State University, Stillwater, OK, United States

**Keywords:** respiratory vaccination, herd health management, producer survey, biosecurity, beef cattle

## Abstract

**Introduction:**

Respiratory disease is a leading cause of death loss among US beef cattle operations and has significant lingering negative impacts on calf health, performance, and financial returns as they move through the supply chain. It can also negatively impact cowherd reproductive performance. Yet, a significant number of beef cattle operations have not adopted respiratory vaccination for calves or the breeding herd.

**Methods:**

This analysis explores the potential reasons why some producers vaccinate their cattle and some do not, including how influential factors regarding vaccination adoption differ between calves and the breeding herd using Probit regression analysis.

**Results:**

Regression results indicate that, for calves, the likelihood of respiratory vaccine adoption is most influenced by herd size and the use of other vaccines. Breeding herd vaccination decisions are more complex, influenced not by herd size but rather by disease knowledge and risk perception, producer education, and cost barriers.

**Discussion:**

Herd health management education efforts through veterinarians and extension services can use these results to better target respiratory vaccination information addressing some of these barriers, improving national cattle herd health.

## 1 Introduction

Bovine respiratory disease (BRD) is the general name of a disease complex shown to have significant negative consequences in U.S. beef operations at all stages of production. It is widely considered the beef industry’s most prevalent and costly disease. Economically, BRD-related losses to livestock producers impose additional costs for treatments and lost revenue due to decreased productivity or animal death loss. The cost of death loss alone in U.S. cattle production was estimated to be $3.87 billion in 2015 with primary losses attributed to non-predator losses such as disease and sickness in cattle (96.2 percent) and calves (84.3 percent) (1).

BRD includes multiple viral and bacterial pathogens, as well as singular parasitic and fungal agents (2). The interactions of these pathogens create disease in the animal. In the cowherd, two BRD viral pathogens, BHV-1 (bovine herpes virus) and BVDV (bovine viral diarrhea virus), have dual effects on respiratory and reproductive disease (3). These two pathogens can decrease herd pregnancy rates via infertility, and increase abortions and congenital defects in offspring, consequently increasing herd health management costs while also negatively impacting the total pounds of cattle marketed (3–5). At the cow-calf level, lightweight calves and particularly those in the weaning transition period are at elevated risk for BRD-related morbidity and mortality with infection rates of up to 10 percent in some herds (2, 6, 7).

The cost of BRD in pre-weaned calves has been estimated at $28 per cow per year on cow-calf operations, substantially impacting profitability (7).Further, the structure of the U.S. beef cattle supply chain is such that health management decisions at the cow-calf level affect growth and performance throughout the beef value chain, most notably at the feedlot level (8). Feedlot environments have a confluence of environmental stressors that predispose cattle to BRD infection. Such stressors may include commingling with other cattle, dust, and rapid weather changes due to regional transportation (6). The economic impact of respiratory disease to a feedlot is estimated to average $23.60 per head fed and is estimated to comprise 7 % of total production costs from the weaning phase to the packer phase in the U.S. cattle industry (2, 9). Feedlot costs are compounded by value lost due to the negative impacts of BRD on carcass quality such as lower USDA quality grades and multiple injection site lesions.

Biosecurity refers to everything that is done to keep diseases and the pathogens that carry them (including viruses, bacteria, fungi, parasites, and other microorganisms) away from livestock, property, and people. Vaccination is an important biosecurity tool in the beef industry for overall herd health management. While vaccines induce varying levels of effective immunity in individual animals, they can effectively improve overall herd immunity and reduce the impacts of clinical illness in the herd, making respiratory vaccination a viable part of a herd health management strategy (10). For example, respiratory vaccines properly utilized in the cow herd have the potential to decrease the reproductive impacts of BHV-1 and BVDV (3–5, 11). Respiratory vaccinations in calves can decrease illness not only at the cow-calf level, but also in subsequent production phases since immune systems are more developed as they move through the supply chain. Vaccination implementation by cow-calf producers may be influenced by herd characteristics, operator attributes, adoption of other biosecurity practices, costs of production, and marketing opportunities (12–16). While various studies have examined vaccination adoption rates in beef cattle, an exploration of the interlacing economic and herd management factors that result in respiratory vaccination adoption for herd health management is lacking in the literature.

The objective of this study was to understand factors affecting Oklahoma cow-calf producers’ respiratory vaccination adoption for the cow herd and for calves. The results highlighted the complexities of respiratory vaccination decisions and indicated that factors of influence differ for cow herd vaccination decisions versus calf crop vaccination decisions. The information garnered from this study may help animal health authorities, veterinarians, and extension educators better target producer education efforts regarding the economic importance of respiratory vaccinations at the cow-calf production phase.

## 2 Prior studies of beef cattle respiratory vaccination adoption

The USDA APHIS National Animal Health Monitoring System reported that Oklahoma’s nonpredator calf death loss rate from respiratory disease (30.0 percent) was higher than the national average (26.9 percent) (17). Results were similar for Oklahoma’s breeding herd with nonpredator death losses due to respiratory disease reported as 26.6 percent compared to 23.9 percent nationally (17). These risks were reflected in respiratory vaccination rates, with 49.0 percent of Oklahoma producers reporting administering at least one round of respiratory vaccinations to calves before marketing compared to 42.5 percent of producers nationally (18, 19). The most common reasons given by producers for not vaccinating calves against respiratory disease in Oklahoma were “I am familiar with the practice, but I do not use it” (49.8 percent) followed by “I have not used this practice in the past, and I have been fine” (13.5 percent) (18).

Vaccination adoption can benefit the cattle producer beyond disease prevention in the cow-calf herd and the calf crop. Previous survey results indicated that nearly 2 out of 5 Oklahoma producers who adopted calfhood respiratory vaccinations did so to participate in a calf health management certification program to capture marketing benefits (20). Superior Livestock reports that the percentage of feeder cattle sold in their cattle auctions through certified health programs increased from 53 percent in 2001 to 88 percent in 2010 (21). Certified health programs increase market prices for feeder calves, with premiums of $2 to $4 per cwt for steers and $1 to $2 per cwt for heifers in addition to any premiums for the individual management practices included in the management bundle (19). Even without certification, respiratory vaccinations can result in price premiums in feeder calf markets (20, 22, 23).

## 3 Materials and methods

### 3.1 Primary survey data

The Oklahoma Cow-Calf Biosecurity Survey was developed by Oklahoma State University through funding from the United States Department of Agriculture, Animal and Plant Health Inspection Service (USDA APHIS), National Animal Disease Preparedness and Response Program (NADPRP). The survey was administered through a contract with the USDA National Agricultural Statistics Service (NASS). The stratified proportionate random sample of 5,000 producers was obtained from the USDA NASS beef cattle list frame and represented a statistically valid sample of the state’s beef cattle producer population. In January 2022, producers received a study selection postcard, followed 2 weeks later by a mail survey with a postage-paid return envelope. Two weeks after the mail survey, producers were contacted via phone with the option to complete the survey over the phone rather than return the paper survey. The data collection process was completed in February 2022. Of the 5,000 producers contacted, 1,466 surveys were completed, either by mail or via phone, resulting in a 29 percent response rate and a 95 percent confidence level associated with the sample.

Screening questions were used to filter out producers who did not actively manage cattle in the 2021 calendar year and those not involved in the cow-calf^1^ production stage. The resulting pool of 981 producers actively managed beef cows and produced calves in 2021. To put this sample in context, the 2022 USDA Census of Agriculture reported 4.5 million beef cattle (2.0 million beef cows) on 43,223 ranching operations (39,338 cow-calf operations) in Oklahoma (24). Oklahoma is a top 5 beef cattle production state, with an average beef cow herd size of 51 beef cows in Oklahoma as compared to a national average of 47 head. Respondents shared producer and cattle operation demographics, current herd management regarding disease testing and vaccinations, knowledge and use of biosecurity elements and the Secure Beef Supply Plan, and disease familiarity and threat perceptions.

Means^2^ for binary variables in Table 1 (e.g., herd size categories) can be interpreted as the percent of the respondents who responded ‘yes’ to the question, and means for continuous variables are the average response to the question across respondents (e.g., the portion of respondents who have not heard the definition of biosecurity). Continuous biosecurity practice variables in Table 2 are mean rates of biosecurity adoption. The local N—or the number of observations remaining after removing respondents that left the question blank when filling out the survey are also included for each variable. All summary statistics are unweighted sample means; no population means were calculated.

**Table 1:** Select 2022 cow-calf biosecurity survey summary statistics.

Survey Question^a^	Variable Name	Mean^b^	N^c^
Cattle operation characteristics			
How many beef cows do you currently manage?	Herd 1–24	0.27	977
	Herd 25–49	0.21	977
	Herd 50–99	0.23	977
	Herd 100–249	0.22	977
	Herd GE 250	0.06	977
Current herd management practices			
In your existing herd, do you test the following groups for BVD-PI (persistently infected) animals?	PI Cows	0.17	907
	PI Bulls	0.23	890
	PI nonbreed	0.08	778
For each practice listed, please indicate whether you do this in your cow-calf operation: Respiratory Vaccinations (RVX) for calves (IBR, BVD, boosters, etc.) prior to marketing?	RVX calves	0.76	943
Respiratory vaccines for breeding herd?	RVX breeding	0.54	866
Keep records of medical treatments (MT) - calves	MT record calves	0.47	934
Keep records of medical treatments - breeding herd	MT record breeding	0.49	900
Clostridial (blackleg) vaccine (CVX) - calves	CVX calves	0.89	937
Clostridial (blackleg) vaccine - breeding herd	CVX breeding	0.63	898
Biosecurity practices and animal movement			
How familiar are you with this definition of biosecurity? Please check the one that most closely applies to you.	Bio not heard	0.31	948
	Bio implemented	0.13	948
	Bio not used	0.56	948
How familiar are you with the recommendations for the Secure Beef Supply Plan (SBS)? Please check only the one that most closely applies to you. *NK = No knowledge; UK = Unknown*	SBS NK UK	0.84	940
	SBS heard used	0.15	940
Disease knowledge			
What is your familiarity with the following diseases present in the United States?	BVD not familiar	0.19	841
	BVD seen the name	0.09	841
	BVD some familiarity	0.23	841
	BVD not in my herd	0.35	841
	BVD in my herd	0.15	841
Before bringing any cattle onto this operation in the last 3 years, did you normally require any vaccination and/or testing for the animals? *Testing only: Johne’s disease, bovine tuberculosis, brucellosis – males and adult females* *Testing, Vaccination, or both: brucellosis – heifers, BVD, respiratory disease (IBR, PI3, BRSV), trichomoniasis, leptospirosis, Other* *Variable Range 0–15.*	Vac-Test	0.45	981
Please rate the threat of introducing the following diseases into your operation (p) due to the arrival of cattle from outside sources (check one): *High threat to moderate threat = Threat* *Low threat to no threat = No Threat* *Unknown threat knowledge = UK*	BRDp threat	0.34	790
	BRDp no threat	0.48	790
	BRDp uk	0.19	790
	BVDp threat	0.33	777
	BVDp no threat	0.46	777
	BVDp uk	0.21	777
Do you believe the following health issues are a significant problem for the beef industry (i)? *High threat to moderate threat = Threat* *Low threat to no threat = No Threat* *Unknown threat knowledge = UK*	BRDi threat	0.58	805
	BRDi no threat	0.19	805
	BRDi uk	0.23	805
	BVDi threat	0.50	788
	BVDi no threat	0.25	788
	BVDi uk	0.26	788
Producer characteristics			
Please circle your age group:	Age LE 44	0.07	981
	Age 45–54	0.11	981
	Age 55–64	0.25	981
	Age 65–74	0.32	981
	Age GE 75	0.19	981
Please circle the category that best describes the highest level of education that you have attained: *High school graduate; Vocational, technical or 2-year degree; Bachelor’s degree; Graduate or professional degree; None of these*	Education > High school	0.58	981
Approximately what percentage of the past year’s household net income (OP) came from your beef cattle operation?	OP income 0 percent	0.11	981
	OP income 1–20 percent	0.43	981
	OP income 21–60 percent	0.24	981
	OP income 61–100 percent	0.07	981

a A full copy of the survey questions will be made available by the corresponding author upon request.

b Percentage of answered variables in Oklahoma.

c Reported percentages are unweighted, sample means.

d N is the subsample observations for each variable.

Source: 2022 OSU Cow-calf Biosecurity Survey.

**Table 2:** Summary statistics across producer response to twenty 2022 cow-calf biosecurity survey^e^ biosecurity plan elements.

Survey Question^a^	Abbreviated name	Mean^b^,^c^	SD^e^	N^d^
For each biosecurity plan element listed in the table below, please indicate whether this practice is used in your cattle farm/ranch.	Bioplan elements $\left(\overset{20}{\underset{i=1}{\Sigma }}\mathrm{Yes}/20\right)$	0.20	0.18	981
For practices where you choose NO, please indicate why you do not use this practice with a checkmark in the box(es) across the row for any and all constraints that apply to you. You may have multiple ✓ or X per row.				
I am not familiar with this practice.	BP not familiar	0.20	0.30	981
I am familiar with this practice but do not use it.	BP do not use	0.07	0.18	981
I have not done this in the past, and things have been okay.	BP been okay	0.07	0.17	981
I do not really know what it requires.	BP uk requirements	0.05	0.14	981
I thought about it. I need help with specifics of how to implement it on my ranch.	BP how to implement	0.00	0.04	981
I sometimes do this, but I have not fully implemented it.	BP not fully implemented	0.01	0.05	981
It is too costly.	BP costly	0.03	0.10	981
It requires too much labor.	BP labor	0.02	0.09	981
I do not have enough cattle to mess with it.	BP cattle	0.17	0.29	981

Source: 2022 cow-calf biosecurity survey.

a A copy of the survey questions will be made available by the corresponding author upon request.

b Percentage of answered Biosecurity Element variables in Oklahoma found from question 3.4 in the 2022 Oklahoma Cow-Calf Biosecurity Survey.

c Reported percentages are unweighted, sample means.

d N is the subsample observations for each variable.

e SD is the standard deviation of the variable.

### 3.2 Probit model

Probit analysis is used when the study population makes a discrete choice. In this case, the discrete choice is the choice to vaccinate against respiratory disease by Oklahoma cow-calf producers. The left-hand side, or dependent variable, of a probit analysis takes the form of 0/1 where 1 indicates that the respondent chose to vaccinate. The coefficients of the right-hand side variables, or independent variables, give insight into the factors that increase or decrease the probability that cow-calf producers would choose to vaccinate. The data were tested for normally distributed errors, and we failed to reject the null hypothesis that errors were normally distributed. In combination with our large sample size, we concluded that the probit model was appropriate for this discrete choice analysis.

This analysis explored independent variables associated with various herd characteristics, production practices, and alternative herd health management practices like biosecurity that may influence vaccination according to the literature. Because different factors may influence respiratory vaccination of breeding cattle in comparison to calves, two separate regressions were developed. The dependent variable for the calf regression is “respiratory vaccination of calves” ( $Calf_{\mathrm{RespVac}}$ ) which takes a value of 1 if the respondent vaccinated and 0 if they did not. Equation 1 does not include breeding herd variables, except the variable indicating whether the breeding herd receives respiratory vaccines. A second regression has the dependent variable “respiratory vaccination of the breeding herd” ( $\mathrm{Breedin}g_{\mathrm{RespVac}}$ ) which takes a value of 1 if the respondent vaccinated and 0 if they did not. Equation 2 includes no calf variables, except the variable indicating whether calves receive respiratory vaccines. The estimated equations are:


(1) $$\begin{array}{c} Calf_{\mathrm{RespVac}}=\beta_{0}+\beta_{1}X_{NE}+\beta_{2}X_{SE}+\beta_{3}X_{SW}\\ +\overset{12}{\underset{n=1}{\Sigma }}\beta_{ED}X_{ED}+\overset{10}{\underset{n=1}{\Sigma }}\beta_{\mathrm{Mgmt}}X_{\mathrm{Mgmt}}\\ +\overset{12}{\underset{n=1}{\Sigma }}\beta_{Bio}X_{Bio}+\overset{10}{\underset{n=1}{\Sigma }}\beta_{\mathrm{Control}}X_{\mathrm{Control}}+\varepsilon \end{array}$$

(2) $$\begin{array}{c} \mathrm{Breedin}g_{\mathrm{RespVac}}=\beta_{0}+\beta_{1}X_{NE}+\beta_{2}X_{SE}+\beta_{3}X_{SW}\\ +\overset{12}{\underset{n=1}{\Sigma }}\beta_{ED}X_{ED}+\overset{10}{\underset{n=1}{\Sigma }}\beta_{\mathrm{Mgmt}}X_{\mathrm{Mgmt}}\\ +\overset{12}{\underset{n=1}{\Sigma }}\beta_{Bio}X_{Bio}+\overset{10}{\underset{n=1}{\Sigma }}\beta_{\mathrm{Control}}X_{\mathrm{Control}}+\varepsilon \end{array}$$

To simplify Equations 1, 2 visually, independent variables are clustered into vectors shown in Table 3, with variable definitions in Tables 1, 2. The vector of variables associated with education and producer knowledge (*X_ED_*) included variables based on producer knowledge of the disease. The administration and herd management (*X_Mgmt_*) vector included variables in which a producer was asked about the administration of vaccinations, testing, and record keeping. The biosecurity vector *(X_Bio_)* has variables based on biosecurity elements and familiarity with the definition of biosecurity and recommendations of the Secure Beef Supply (SBS) Plan. Finally, a vector of control variables other than geographic region was included (*X_Control_*). Regional variables (*X_NE_, X_SE_, X_SW_*) control geographic variability with the Northwest region in the intercept. We included regional control variables for differences in weather and ecosystems across the state. The intercept is denoted by β_0_.

**Table 3:** Variable categories for probit regression in Equations 1, 2.^a^

Knowledge (ED)	Administration (Mgmt)	Biosecurity (Bio)	Control
BVD seen	PI Cows	Bio not heard	Herd 25–49
BVD some familiar	PI Bulls	Bioplan_elements	Herd 50–99
BVD not in my herd	PI Nonbreeding	BP__uk_requirements	Herd 100–249
BVD in my herd	RVX breeding (Equation 1 only)	BP_been_okay	Herd GE 250
BRDp Threat	RVX calves (Equation 2 only)	BP_cattle	Age 55–64
BRDp UK	CVX calves	BP_costly	Age 65–74
BVDp Threat	CVX breeding	BP_dont_use	OP income 1–20 percent
BVDp UK	Vac-Test	BP_how_to_implement	OP income 21–60 percent
BRDi Threat	MT record calves	BP_labor	OP income 61–100 percent
BRDi UK	MT record breeding	BP_not_familiar	ED higher HS
BVDi Threat		BP__not_fully_implemented	
BVDi UK		SBS_heard_used	

a Variables came from the 2022 cow-calf biosecurity survey.

A probit model regression gives estimated coefficients ( $\beta_{1},\beta_{2},\beta_{3},\beta_{ED},\beta_{\mathrm{Mgmt}},\beta_{Bio},\beta_{\mathrm{control}})$ in the form of z-scores, which can be hard to interpret into something meaningful. Transforming coefficients of the regression into marginal values using the *margins* function in R resulted in coefficients that are easier to interpret. Marginal values are partial derivatives of the regression with regard to the other variables. In other words, marginal effects illustrate how a small change in one independent variable, holding other independent variables constant, affected the probability of a producer vaccinating either calves or the breeding herd. These margins were interpreted as predicted probabilities (25). Results discussion focuses on the sign of the marginal value (i.e., did it increase or decrease the predicted probability of vaccination) and the magnitude (i.e., did it increase the predicted probability more or less as compared to other independent variables). Error terms (*ε*) were assumed to be normally distributed in probit models.

## 4 Results

### 4.1 Preliminary analysis of survey data

**Figure 1:**
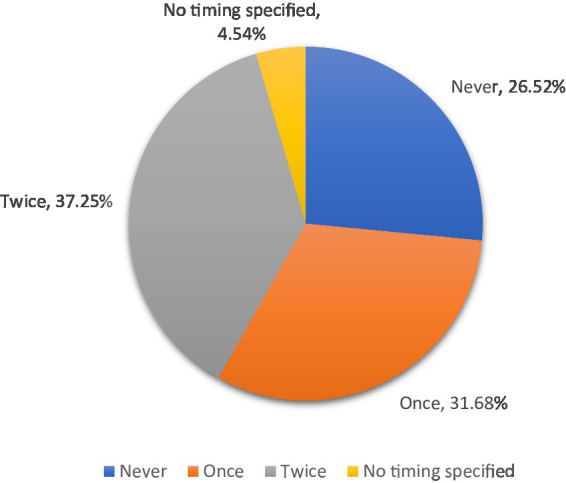
Frequency of calfhood respiratory vaccinations administered prior to marketing, 2021. Calculated by the authors using 2022 cow-calf biosecurity survey data. Percentage of the Oklahoma respondents that answered the question “Please indicate whether you do this [practice] for your cow-calf operation – Respiratory vaccines for calves (IRB, BVD, boosters, etc.) prior to marketing.” Then followed up with “If yes, how many rounds?”

**Figure 2:**
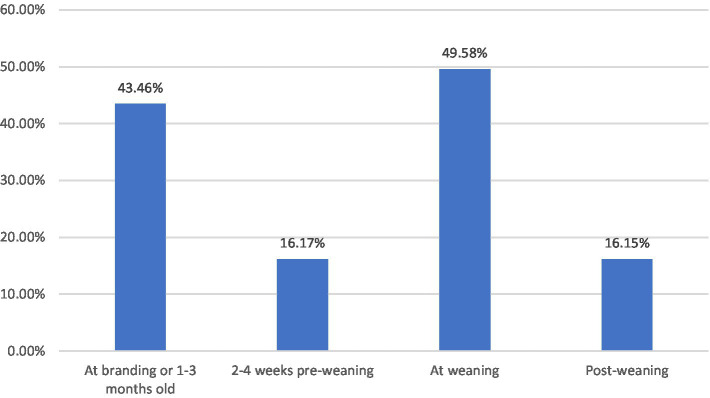
Producer-reported administration of multiple respiratory vaccines, 2021. Calculated by the authors using 2022 cow-calf biosecurity survey data. Percentage of the Oklahoma respondents that answered the question “Please indicate whether you do this [practice] for your cow-calf operation – Respiratory vaccines for calves (IRB, BVD, boosters, etc.) prior to marketing.” Then respondents selected one or more of the timing options “At branding or 1–3 months old,” “2–4 weeks pre-weaning,” “at weaning,” or “post-weaning”.

Respiratory vaccination for calves was utilized by 73.5 percent of respondents (Figure 1). Most producers in the state vaccinated calves twice (37.3 percent of all respondents), and the most common timing was “at branding or 1 to 3 months old” (43.5 percent) and “at weaning” (49.6 percent) (Figure 2). The breeding herd was vaccinated by 47.9 percent of respondents. Of those who vaccinated the breeding herd 34.4 percent used a killed vaccine and 21.3 percent used a modified live vaccine (26.5 percent of respondents did not specify vaccine type). Variations in weather, stocking rates, and cattle movements across the state likely influenced the risk of respiratory disease.

**Figure 3:**
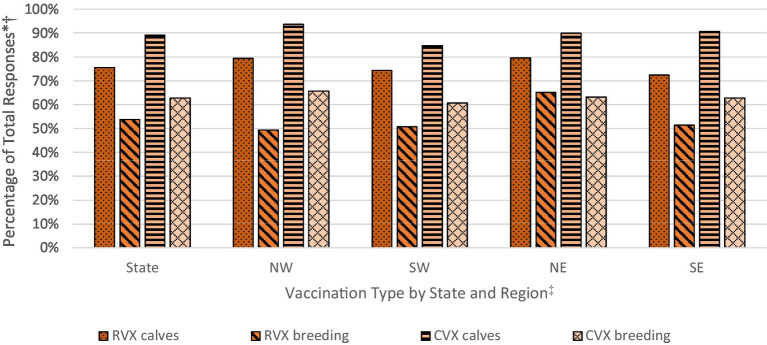
Respiratory and clostridial vaccination rates for calves and breeding herd, statewide and by region. Calculated by the authors using 2022 cow-calf biosecurity survey data. * Percentage of total respondents in Oklahoma that responded to the use of respiratory (RVX) and clostridial (CVX) vaccination for calves and/or for the breeding herd in the survey. † Reported percentages are unweighted, sample means. ^‡^Regions are broken by Interstate I-35 (east/west) and Interstate I-40 (north/south) to create regions for the northwest (NW), southwest (SW), northeast (NE), and southeast (NE).

Differences in vaccination rates by vaccination type (RVX for respiratory vaccines and CVX for clostridial vaccines) and animal type (‘calves’ and ‘breeding’ for all beef cows, bulls and weaned replacement heifers) reveal two patterns (Figure 3). First, calves were vaccinated at a higher rate than the breeding herd for both respiratory (75.6 percent *RVX calves* and 53.8 percent *RVX breeding*) and clostridial diseases (89.1 percent *CVX calves* and 62.8 percent *CVX breeding*). Second, clostridial vaccinations for calves and the breeding herd were implemented at a noticeably higher rate than respiratory vaccinations. Regionally, the northern half of the state had higher rates of respiratory vaccination. Within this context, factors affecting calf and breeding herd vaccination rates were explored.

A somewhat unique characteristic of the state is that some cow-calf producers retain calves after weaning to gain value by grazing on small grains—primarily hard red winter wheat—in the winter. Selling calves at weaning was still the most common (55.2 percent of respondents), but 35.2 percent grazed for 30 days post-weaning and 19.4 percent retained calves through the stocker/backgrounding stage. Retaining calves through finishing and selling direct beef to consumers (freezer beef) was becoming more common, with 14.8 percent of respondents routinely participating in this activity in the last 5 years. Biosecurity needs may be different for producers bringing additional calves in to graze in the winter as compared to only grazing their own calves or selling calves at weaning.

Testing and vaccination results indicated that testing rates for BVD-PI animals are relatively low for cows (16.8 percent), bulls (22.7 percent), and non-breeding stock (8.2 percent) (Table 1). Vaccination can be combined, in a variety of ways, with testing before new herd additions enter the farm. Producers were asked to report whether they test for Johne’s disease, bovine tuberculosis, brucellosis (males and adult females), and whether they use testing, vaccination, or both for brucellosis (heifers), BVD, respiratory disease (IBR, PI3, BRSV), trichomoniasis, and leptospirosis. There were a total of 15 testing or vaccination combinations. The *Vac-Test* variable in Table 1 was the average number of vaccination and testing practices for respiratory disease that the producer required. The average value of 45 percent indicated that a producer desired between 6 and 7 vaccines and/or tests from the list above. Record keeping for herd medical treatment was reported in 47.3 percent of operations for calves and 49.0 percent of operations for the breeding herd (Table 1).

Perceived risk associated with disease has been another reason to adopt vaccination programs. Producers were asked to assess the threat of BRD, and more specifically BVD on their own operation. BVD was anticipated to be the most currently discussed viral disease of the BRD complex by producers in Oklahoma. Results were groups into those that felt BRD was a moderate to large threat (*BRDp threat*), a low threat or no threat *(BRDp no threat)*, or an “unknown” threat level (*BRDp uk*). Similar questions were asked regarding specific producer perceptions about perceived BVD threat levels (*BVDp threat, BVDp no threat, BVDp uk*). For both BRD and BVD, a higher percentage of producers did not perceive them as a threat to their operation (47.6 percent *BRDp no threat* and 45.8 percent *BVDp no threat*).

A second series of questions asked about the threat of BRD, and BVD specifically, to the beef industry in general. Producers perceived disease to be a greater threat to the industry (58.0 percent *BRDi threat* and 49.6 percent *BVDi threat*) in general than to their own cow-calf operation, which may have reflected the large losses experienced in the feedlot sector.

Understanding that both calf and cow herd vaccinations are a part of comprehensive biosecurity plans, producers were provided with the definition of biosecurity. As stated in the introduction, for this survey, biosecurity referred to everything that was done to keep diseases and the pathogens that carry them (including viruses, bacteria, fungi, parasites, and other microorganisms) away from livestock, property, and people. Statewide (Table 1), most producers had either not heard of the definition (30.7 percent, *Bio not heard*) or had heard of it but had not implemented biosecurity into their operation (56.0 percent, *Bio not used*). Only 13.3 percent of producers had implemented some level of biosecurity into their operation (*Bio implemented*). Producers were also asked about their familiarity with the recommendations of the Secure Beef Supply Plan. Only 15.4 percent of the producers had heard of the Secure Beef Supply Plan (*SBS heard used*) and had some level of implementation of it in their operation. Adoption rates represent what producers’ reported adoption of specifically described biosecurity practices for the breeding herd and for the calf crop. It is noted that some producers potentially incorporated management practices with biosecurity benefits for other reasons such as profitability, without necessarily acknowledging the biosecurity benefits fully. For instance, producers may have vaccinated cattle for cost reduction or to capture market premiums without full understanding of the biosecurity implications.

The SBS or Beef Quality Assurance (BQA) guidelines for a written biosecurity plan were broken down into 22 individual activities for this survey. Producers were asked to indicate biosecurity activity implementation (yes = 1, no = 0) on their operation. A percentage of biosecurity plan activities adopted by each respondent was calculated (Table 2). Across all respondents, only 4 biosecurity plan activities were adopted by more than half of the producers; this corresponds to an average proportion of biosecurity plan adoption of 18.2 percent (*Bioplan Elements*). Further, if a producer answered “no” for a particular activity, they were asked to select one of 9 reasons why they did not adopt it. The most common reason an activity was not adopted was due to lack of familiarity with the activity (*BP not familiar*), followed by the producer feeling they did not have enough cattle (*BP cattle*) to make it worthwhile.

Finally, a typical producer in the state was between the age of 65–74, received 1 to 20 percent of their household income from the cattle operation, and had an education level higher than a high school degree (Table 1). Demographics were used to control for inherent differences between producers.

### 4.2 Probit results

Probit analysis was completed using the statistical software R. Due to the large number of independent variables, statistical checks were incorporated. First, model fit was examined using the Bayesian Information Criterion (BIC) and Akaike Information Criterion (AIC). The final calf vaccination regression included 638 local observations and 41 variables. While this is a large number of variables, it resulted in the best model specification fit statistics. The breeding herd regression included 526 local observations and 45 variables. Second, variance inflation factors (VIF) were examined to assess the correlation between independent variables, with no variables included in the final regression exhibiting VIF values over 4. Both models were statistically significant based on Likelihood Ratio Tests and Wald tests.

As noted earlier, our focus in this results discussion will be on the sign and magnitude of each marginal effect relative to other marginal effects. There were only five variables of statistical significance (Table 4, in bold), all with a positive effect on a producer’s decision to vaccinate their calves for respiratory disease. Clostridial vaccination of calves, respiratory vaccinations of the breeding herd, and larger herd sizes were all significant, positive influencers on a producer’s decision to vaccinate calves against respiratory disease. The use of clostridial vaccinations in the calves increased the predicted probability that a producer vaccinated their calves for respiratory disease by 0.2421. The use of respiratory vaccination on the breeding herd increased the predicted probability that a producer vaccinated their calves for respiratory disease by 0.2131. The probability of vaccinating calves increased with herd size. A herd size of 50 to 99 head increased the predicted probability of calf respiratory vaccination by 0.1244 as compared to those with very small (1 to 25 head) herds. Likewise, a herd size of 100 to 249 head increased the predicted probability that calves received respiratory vaccination by 0.1367, and herd sizes of 250 head or more increased the predicted probability by 0.1789 compared to a herd of 1 to 25 head.

**Table 4:** Marginal values for probit regression of calf respiratory vaccination administration.

Variable	AME^a^	SE	*Z*-score	*p*-value^b^	Confidence interval	
					Lower	Upper
Age 55 to 64	0.05	0.03	1.38	0.17	−0.02	0.11
Age 65 to 74	−0.01	0.03	−0.24	0.81	−0.07	0.05
bio_not_heard	0.01	0.03	0.45	0.65	−0.05	0.08
Bioplan_elements	0.02	0.09	0.26	0.80	−0.15	0.19
BP__uk_requirements	−0.09	0.08	−1.08	0.28	−0.25	0.07
BP_been_okay	−0.05	0.07	−0.68	0.50	−0.18	0.09
BP_cattle	0.00	0.05	0.10	0.92	−0.09	0.10
BP_costly	0.19	0.17	1.13	0.26	−0.14	0.52
BP_dont_use	−0.04	0.08	−0.46	0.65	−0.19	0.12
BP_how_to_implement	2.13	1.33	1.61	0.11	−0.47	4.73
BP_labor	−0.17	0.16	−1.05	0.29	−0.49	0.15
BP_not_familiar	−0.06	0.04	−1.38	0.17	−0.15	0.03
BP_not_fully_implemented	0.13	0.29	0.44	0.66	−0.44	0.70
BRDi_threat	0.00	0.04	0.03	0.97	−0.08	0.09
BRDi_uk	0.02	0.05	0.36	0.72	−0.08	0.12
BRDp_threat	0.02	0.04	0.55	0.58	−0.06	0.11
BRDp_uk	0.03	0.05	0.67	0.51	−0.06	0.13
BVD_in_my_herd	0.02	0.05	0.29	0.77	−0.09	0.12
BVD_not_in_my_herd	0.01	0.04	0.15	0.88	−0.07	0.09
BVD_seen	0.05	0.05	1.09	0.27	−0.04	0.15
BVD_some_familiar	0.04	0.04	0.88	0.38	−0.05	0.12
BVDi_threat	−0.01	0.04	−0.26	0.79	−0.09	0.07
BVDi_uk	−0.06	0.05	−1.13	0.26	−0.16	0.04
BVDp_threat	0.06	0.04	1.41	0.16	−0.02	0.15
BVDp_uk	−0.05	0.05	−0.97	0.33	−0.14	0.05
**cvx_calves**	**0.24**	**0.04**	**6.31**	**0.00**	**0.17**	**0.32**
ed_higher_hs	0.02	0.03	0.88	0.38	−0.03	0.08
**herd100to249**	**0.14**	**0.04**	**3.07**	**0.00**	**0.05**	**0.22**
herd25to49	0.05	0.04	1.30	0.19	−0.02	0.12
**herd50to99**	**0.12**	**0.04**	**3.32**	**0.00**	**0.05**	**0.20**
**herdGE250**	**0.18**	**0.08**	**2.31**	**0.02**	**0.03**	**0.33**
mt_record_calves	0.02	0.03	0.72	0.47	−0.03	0.07
op_income_1to20percent	0.02	0.03	0.71	0.47	−0.04	0.09
op_income_21to60percent	−0.06	0.04	−1.42	0.16	−0.14	0.02
op_income_61to100percent	−0.06	0.06	−0.97	0.33	−0.18	0.06
region_ne	−0.06	0.04	−1.41	0.16	−0.14	0.02
region_se	−0.06	0.04	−1.53	0.13	−0.13	0.02
region_sw	0.00	0.04	0.07	0.95	−0.07	0.08
**rvx_breeding**	**0.21**	**0.03**	**8.33**	**0.00**	**0.16**	**0.26**
sbs_heard_used	0.01	0.04	0.19	0.85	−0.07	0.09
vac_test	0.03	0.02	1.55	0.12	−0.01	0.06

Source: author estimates based on the 2022 cow-calf biosecurity survey.

a Probit regression results for respiratory vaccinating the breeding herd in the form of marginal values. Please refer to Table 1 for specific variable definitions.

b Bold text indicates statistical significant variables.

Shifting to breeding herd results, respiratory vaccination of calves, clostridial vaccination of the breeding herd, keeping medical records on the breeding herd, education, producer perception of BRD in the industry, and a producer’s decision to not adopt biosecurity plan elements due to cost were all significant influences on a producer’s decision to vaccinate their breeding herd for respiratory disease (Table 5). The use of respiratory vaccinations in calves increased the predicted probability of vaccinating the breeding herd for respiratory disease by 0.3783. The use of clostridial vaccinations in the breeding herd increased the predicted probability of also vaccinating them for respiratory disease by 0.1714. A producer who kept written medical records on the breeding herd had an increased predicted probability of vaccinating their breeding herd for respiratory disease by 0.0919. Producers with a secondary educational degree had a higher predicted probability of vaccinating their breeding herd for respiratory disease by 0.0899.

**Table 5:** Marginal values for probit regression of breeding herd respiratory vaccination administration.

Variable	AME^a^	SE	*Z*-score	*p*-value^b^	Confidence interval	
					Lower	Upper
Age 55 to 64	−0.04	0.04	−0.84	0.40	−0.12	0.05
Age 65 to 74	−0.02	0.04	−0.58	0.57	−0.10	0.06
bio_not_heard	−0.01	0.05	−0.27	0.79	−0.10	0.08
Bioplan_elements	0.11	0.11	0.99	0.32	−0.11	0.34
BP__uk_requirements	−0.05	0.11	−0.47	0.64	−0.27	0.17
BP_been_okay	0.01	0.09	0.14	0.89	−0.16	0.19
BP_cattle	−0.03	0.07	−0.50	0.62	−0.17	0.10
**BP_costly**	**−0.65**	**0.21**	**−3.15**	**0.00**	**−1.05**	**−0.24**
BP_dont_use	0.18	0.10	1.85	0.06	−0.01	0.37
BP_how_to_implement	0.01	0.45	0.02	0.98	−0.87	0.89
BP_labor	0.38	0.26	1.44	0.15	−0.14	0.89
BP_not_familiar	0.01	0.06	0.19	0.85	−0.11	0.14
BP_not_fully_implemented	−0.45	0.34	−1.32	0.19	−1.12	0.22
BRDi_threat	−0.04	0.06	−0.64	0.52	−0.15	0.08
**BRDi_uk**	**−0.19**	**0.08**	**−2.45**	**0.01**	**−0.34**	**−0.04**
BRDp_threat	0.01	0.05	0.16	0.87	−0.10	0.12
BRDp_uk	0.03	0.08	0.43	0.67	−0.12	0.18
BVD_in_my_herd	0.10	0.07	1.34	0.18	−0.04	0.23
BVD_not_in_my_herd	0.05	0.06	0.82	0.41	−0.07	0.16
BVD_seen	−0.09	0.07	−1.24	0.22	−0.24	0.05
BVD_some_familiar	−0.03	0.06	−0.41	0.68	−0.15	0.10
BVDi_threat	0.10	0.05	1.89	0.06	0.00	0.21
BVDi_uk	0.13	0.07	1.77	0.08	−0.01	0.28
BVDp_threat	−0.11	0.06	−1.88	0.06	−0.22	0.00
BVDp_uk	−0.05	0.08	−0.69	0.49	−0.20	0.10
**cvx_breeding**	**0.17**	**0.03**	**4.99**	**0.00**	**0.10**	**0.24**
**ed_higher_hs**	**0.09**	**0.04**	**2.47**	**0.01**	**0.02**	**0.16**
herd100to249	0.09	0.06	1.58	0.12	−0.02	0.21
herd25to49	0.08	0.05	1.47	0.14	−0.03	0.18
herd50to99	0.06	0.05	1.20	0.23	−0.04	0.17
herdGE250	0.07	0.08	0.83	0.40	−0.09	0.22
**mt_record_breeding**	**0.09**	**0.04**	**2.59**	**0.01**	**0.02**	**0.16**
op_income_1to20percent	−0.07	0.05	−1.51	0.13	−0.17	0.02
op_income_21to60percent	−0.05	0.05	−0.84	0.40	−0.15	0.06
op_income_61to100percent	0.00	0.08	0.02	0.98	−0.15	0.15
PI_bulls	−0.01	0.06	−0.09	0.93	−0.13	0.12
PI_cows	0.11	0.09	1.23	0.22	−0.06	0.28
PI_nonbreed	0.09	0.09	1.06	0.29	−0.08	0.26
region_ne	0.09	0.05	1.74	0.08	−0.01	0.20
region_se	0.06	0.05	1.25	0.21	−0.04	0.17
region_sw	0.07	0.05	1.44	0.15	−0.03	0.17
**rvx_calves**	**0.38**	**0.04**	**8.66**	**0**	**0.29**	**0.46**
sbs_heard_used	0.00	0.05	0.05	0.96	−0.09	0.09
vac_test	0.01	0.02	0.36	0.72	−0.03	0.05

Source: author estimates based on the 2022 cow-calf biosecurity survey.

a Probit regression results for respiratory vaccinating the breeding herd in the form of marginal values. Please refer to Table 1 for specific variable definitions.

b Bold text indicates statistical significant variables.

However, if a producer did not know what BRD was, the predicted probability of vaccination declined. If a producer responded “unknown” when asked to what extent BRD is a threat to the industry, the predicted probability of vaccinating their breeding herd for respiratory disease decreased by 0.1895. In addition, if a producer found biosecurity plan adoption to be too expensive, the predicted probability of vaccination adoption in the breeding herd declined by 0.6463. Overall, this suggests that a producer who participated in some good herd health management practices, including biosecurity practices, was more likely to vaccinate their breeding herd for respiratory disease.

## 5 Implications for the economic impact of respiratory disease

Management of herd health at the cow-calf level impacts individual animal and herd health at the cow-calf stage; however, it also affects the downstream health of calves at the stocker/backgrounding and feedlot levels, eventually affecting the availability and quality of beef at the retail level. Most of the beef produced in the U.S. is high value, grain fed beef; much of that beef is consumed domestically. However, in 2024 the U.S. was projected to export 1.4 million metric tons of beef, accounting for over 11 percent of global beef exports (26). As a result, animal health improvements or declines in the U.S. can have implications across the world for beef prices and availability.

Domestically, strides in reducing the 20 percent prevalence of BRD in feedlots could benefit beef processors and consumers, although cow-calf producers do not receive much benefit and could experience a price decline (27). While Johnson and Pendell (27) and other studies have found that the stocker/backgrounding and feedlot industries benefit from vaccination at the cow-calf level, studies have not shown the same benefit to cow-calf producers.

Cow-calf producers must see benefits on their own operation from respiratory vaccination to incentivize implementation at levels higher than those reported here (73.5 percent for calves and 47.9 percent for breeding cattle). For calves, higher vaccination rates could be driven by market benefits. Calf health management certification programs, like the Oklahoma Quality Beef Network, have historically drawn vaccination premiums from buyers of more than $1.44 per hundredweight, with an additional premium of up to $4.86 per hundredweight for certification that vaccination has been bundled with recommended weaning periods that enhance vaccine effectiveness downstream (23). The motivations for vaccinating the breeding herd are more complex, because the economic benefits are less direct. Prevention of an abortion or weak calf born on the ranch has added value for the producer, but not the same visible benefit as reduced death loss in calves after birth. This study reveals the relationship between respiratory disease knowledge, management activities, and other herd health management practices such as testing and biosecurity for influencing vaccination adoption in the breeding herd.

Since clostridial (blackleg) vaccination rates are higher in both calves and the breeding herd, and producers that administer blackleg vaccines are more likely to administer respiratory vaccines, increasing education on herd vaccination plans may be beneficial. Further, producers who maintain medical records are more likely to administer respiratory vaccinations to the breeding herd, perhaps pointing to a greater awareness of the losses associated with treating illness. Improved overall disease risk understanding in the cow-calf sector facilitates better-informed herd health decisions. Reduced disease costs resulting from the BRD complex justifies producer investment in breeding herd and calfhood vaccinations.

## 6 Conclusion

This study explored the factors that motivated higher rates of respiratory vaccination adoption among cow-calf producers, for both calf vaccination and breeding herd vaccination. The unique survey data and robust response allowed several new variables to be explored compared to the previous literature. Multiple factors were included in the regression that could have influenced vaccination adoption, but the final set of significant coefficients aligned well with the practices producers have received market incentives to adopt—as in the OQBN program or other post-weaning conditioning programs. Further, the gap between vaccination adoption and understanding of biosecurity highlighted an opportunity for improved producer education. With a high percentage of producers not familiar with the term ‘biosecurity’, it is important that messaging particularly that from Extension, veterinarians, and industry be directed at this audience and plain language be used in educational materials. Future work could explore the financial benefits and costs of implementing vaccination programs at the cow-calf level. Market premiums such as the $1.44 per hundredweight bonus received from selling vaccinated cattle at an OQBN sale could benefit producers when they sell their calves (23). One limitation of the biosecurity survey data used in this study was the lack of questions regarding the costs of health management in the herd. As a consequence, producer costs of vaccination could not be included in the analysis.

The results of this study can be used by bovine veterinarians in both private and public practice to target respiratory vaccine education and biosecurity education in the cow-calf sector. Such programs have proven to be effective. A comparison of 2022 survey results to a 2017 state-level Beef Management and Marketing survey, showed an increase of nearly 7 percent in calfhood respiratory vaccination rates (20). This increase is encouraging given the educational efforts by veterinarians and university Extension educators promoting vaccination over the last 5 years. According to the surveys, there are also double the number of producers in 2022 testing their cows for BVD-PI than the producers in 2017 (20).

Vaccination and other herd health management practices are complicated decisions for producers and are influenced by several decision factors. This study shines new light on the reasons for vaccination adoption in Oklahoma and motivates additional research on the topic for the cow-calf sector.

## Data Availability

The datasets presented in this article are not readily available because data were collected by USDA National Agricultural Statistics Service and are protected under the guidelines of Confidential Information Protection and Statistical Efficiency Act (2002). Requests to access the datasets should be directed to SM.NASS.Data.Lab@usda.gov.

## Footnotes

^1^In this study, “cow-calf” production includes any cattle operation that has breeding beef cows and calves at least through weaning. Cow-calf producers may retain calves through background grazing or even feeding, but they must manage a beef cow herd to be included in this analysis.

^2^USDA NASS cleared all summary statistics and regression results to assure data confidentiality was maintained in the process of this analysis.
